# Management of Endocrinopathies During Pregnancy: A Systematic Review

**DOI:** 10.7759/cureus.70554

**Published:** 2024-09-30

**Authors:** Zlatko Kirovakov, Elitsa Gyokova, Nadezhda Hinkova, Boris Stoilov

**Affiliations:** 1 Department of Midwifery Care, Faculty of Health Care, Medical University – Pleven, Pleven, BGR; 2 Department of Obstetrics and Gynecology, Faculty of Medicine, Medical University - Pleven, Pleven, BGR; 3 Department of Obstetrics and Gynecology, University Hospital Saint Marina - Pleven, Pleven, BGR; 4 Department of Obstetrics and Gynecology, Medical University of Plovdiv, Plovdiv, BGR

**Keywords:** endocrinopathies, gdm, glyburide, glycohemoglobin (hba1c), metformin, pregnancy, pregnancy-induced hypertension (pih)

## Abstract

Uncertainty surrounds the efficacy and security of several medications in treating endocrinopathies, such as gestational diabetes mellitus (GDM) in individuals whose normal glucose levels cannot be maintained by diet and exercise alone. To improve pregnancy results for GDM individuals, the present review is conducted to measure the effectiveness of several antidiabetic medications for glucose management. Up until 2024, we looked through PubMed and Google Scholar. Patients with GDM were enrolled in randomized controlled studies that examined several medications. Using the Cochrane risk of bias method, we obtained the pertinent data and evaluated the bias probability. To determine the odds ratio and the surface of the cumulative ranking function of the maternal and neonatal consequences of various therapies in GDM individuals, we first performed pair-wise meta-assessments and subsequently used a systematic review. Macrosomia, higher gestational ages, infant hypoglycemia, and birth weight are the neonatal outcomes. Glycohemoglobin (HbA1c), and pregnancy-induced hypertension (PIH) are the maternal outcomes. This thorough analysis of 25 trial designs found that metformin had fewer cases of macrosomia, higher gestational ages, infant hypoglycemia, and decreased birth weight when compared to glyburide. Metformin was found to be the fastest way to control blood sugar levels in individuals with GDM, whereas glyburide was found to be the most successful medicine for the same purpose.

## Introduction and background

Endocrine illnesses and their treatment for the duration of pregnancy are significant topics for healthcare professionals, diabetes specialists, obstetricians, gynecologists, along various medical specialists involved because of their possible influence on pregnancy and fetal development. It is a transitory structure that plays a significant part in the growth of the maternal-fetal unit and the subsequent appearance of several endocrine events. Throughout pregnancy, the hormonal physiology of both the mother and the fetus changes continuously. Both the mother and the fetus adjust to this growth throughout pregnancy using different processes, such as modifications to their endocrine systems and associated feedback modification [1]. During the first stage of pregnancy, the majority of the hormonal glands begin producing hormones; therefore, the fetus's digestive system is entirely dependent on the mother. The fetal glands continue growing until birth, both in terms of function and form, but the fetus is no more as reliant on the mother's endocrine system following pregnancy [2]. Clinical settings requiring endocrinology medication while pregnant relate to two distinct conditions: treating symptoms or diseases discovered during pregnancy or following treatment for a determined endocrine disease recognized before pregnancy. Depending on the stage of gestation, drug treatment throughout the pregnancy exposes the fetus and mother to risks as well as serious side effects [3].

Clinical studies while pregnant are rare, dangerous, and expensive, necessitating rigorous ethical guidelines and long-lasting follow-up. The developing baby is bound to an all-or-nothing law during the first two weeks of pregnancy, which means that medicine could either cause embryonic mortality or have no effect at all on the advancement of the pregnancy. Differentiation of cells and organ development takes place throughout the next eight weeks, or the major and minor organogenesis process period, which ends at the end of the first period. Any drug assumed at this time needs to be confirmed to have no teratogenic risk and not cause birth defects [4]. The medications may cause fetal toxicity in the second and third trimesters, particularly in the central nervous system [5].

Endocrinologists and obstetricians have therapy challenges when managing hormonal imbalances during pregnancy because of the possible harm they might cause to the health of the mother and fetus [6]. The placenta's role and the connection between the mother and fetus are responsible for the physical and endocrine changes that occur during pregnancy. In the second trimester of pregnancy, fetal endocrine gland development and hormone secretion are finished [7]. Fetal hormonal demands before this signature are entirely dependent on the mother, which is significant during pregnancy. In pregnancy, thyroid conditions, calcium and vitamin D metabolism, and glucose metabolism are endocrine factors that need to be addressed [8,9]. Previous and gestation-induced hormonal imbalances can be used to describe the corresponding conditions. Because endocrine diseases and normal pregnancies share many symptoms, diagnosing them can be challenging [10]. Worldwide, approximately one in six pregnancies is affected by diabetes during pregnancy. The hyperglycemia form detected for the first time in the duration of pregnancy is mentioned as gestational diabetes mellitus (GDM). GDM can arise at any moment for the duration of pregnancy; however, it’s most commonly recognized afterward the 24th week of gestation due to comprehensive regular glucose screening not being accessible for women of reproductive age before pregnancy or throughout the first trimester [11].

GDM is characterized by a diagnosis of diabetes made during the second or third trimester of pregnancy in the absence of overt diabetes. Treatment recommendations for GDM include dietary changes, physical activity, and pharmaceutical interventions such as insulin or antidiabetic medications taken by oral. GDM affects 4-12% of pregnancies and is one of the most prevalent endocrine illnesses among pregnant women [12]. It develops as a consequence of both the hormonal changes that accompany pregnancy and physiological adaptation problems to insulin resistance [13]. Significant associations have been established between GDM and adverse feto-maternal and newborn consequences, including macrosomia, which is delivery prematurely, as well as small for gestational age [14]. GDM represents a pregnancy-specific condition described as diabetes that initially becomes apparent in the second or third month of pregnancy, which is obviously not explicit diabetes (by employing revised terminology by the standards of the American Diabetes Association, 2021) [15-17]. This condition encompasses GDM-1 (managed using diet) and GDM-2 (where needed, managed by incorporating insulin into the diets to correct glycemic levels). GDM is a significant public healthcare concern. Globally, 16% average of pregnancies acquire GDM (equivalent to 18 million women) based on the location and diagnosis [18,19] and between 80 and 90 percent of instances acquire GDM-1 (when dietary intervention is adequate to restore glycaemia). This condition is linked to long-term and short-term (perinatal) problems in both infant and mother. Long-term problems comprise a higher likelihood of acquiring type two diabetes and obesity late in life for both the infant and the mother, demonstrating GDM's negative hereditary repercussions [20]. This is consistent with the paradigm of the Developmental Origins of Health and Disease (DOHaD) [21]. Early detection of GDM risks enables timely therapies to decrease maternal and infant problems [22,23]. Obesity, GDM in a previous pregnancy, older mothers, ethnicity, and multi-parity, along with a family record of obesity, all contribute to GDM incidence [24-26].

The goal of this systematic review is to improve pregnancy results for GDM individuals and performed to assess the effectiveness of several antidiabetic medications for glucose management.

## Review

Material and Methods

Search Strategy

The present systematic review conducted a search of databases such as PubMed and Google Scholar was conducted from 2019-2024. GDM, metformin, glyburide, insulin, macrosomia, higher gestational ages, infant hypoglycemia, birth weight, glycohemoglobin (HbA1c), and pregnancy-induced hypertension (PIH) are the following keywords utilized to search.

Eligibility Criteria

The articles were selected based on predetermined inclusion and exclusion criteria.

The inclusion criteria for the review were as follows: studies involving pregnant women diagnosed with gestational diabetes mellitus (GDM) were considered. The studies needed to specifically examine the use of antidiabetic medications, including metformin, glyburide, and insulin, as part of the management of GDM. Additionally, the studies had to report on both neonatal and maternal outcomes, such as macrosomia, higher gestational ages, infant hypoglycemia, birth weight, HbA1c levels, and pregnancy-induced hypertension (PIH). Only randomized control trials (RCTs) were included in this review. Furthermore, the studies were required to be published in English and within the timeframe from 2019 to 2024.

The exclusion criteria for the review were as follows: studies involving women with pre-existing diabetes diagnosed before pregnancy were excluded. Additionally, studies that focused on non-pregnant individuals were not considered. Studies that failed to report on important maternal or neonatal outcomes or provided insufficient detail for data extraction necessary for meta-analysis were excluded. Non-randomized studies, case reports, and observational studies were also not eligible for inclusion. Any studies published before 2019, as well as those published in languages other than English without a suitable translation, were excluded. Finally, studies that exhibited an elevated risk of bias, including those with inadequate randomization, a lack of blinding, or substantial losses to follow-up without appropriate analysis, were also excluded from the review.

Data Extraction

Data collected from every chosen study have been extracted utilizing a data extraction framework. For every qualified study, the data extracted were as follows: the first author's name, journal title, publication year, research country, method, population as well as samples, health and demographic-related features, including body mass index, age group, and approaches utilized for the GDM screening and related values, which includes blood sugar testing.

Analyzing the Bias Risk

In the risk of bias, the Cochrane risk of bias method was used to evaluate the bias probability in the included studies. Every study has been evaluated for selection, performance, attrition, reporting, and other potential sources of biases based on the standard provided within the Cochrane Risk of Bias tool for systematic reviews of interventions.

Outcome Measures

The outcomes of interest have been separated into two groups. They are maternal and neonatal consequences. Macrosomia, higher gestational ages, infant hypoglycemia, and birth weight are the neonatal outcomes. HbA1c and PIH are maternal outcomes.

Statistical Analysis

In the statistical analysis, we performed a pairwise meta-analysis using a fixed-effects or randomized-effects approach. The fixed-effect meta-analysis approach has been utilized for merging the data from separate trials within the lack of substantial heterogeneity. In an occurrence of substantial heterogeneity, the random effect approach has been employed. For continuous variables, the standardized mean difference (SMD) has been computed as the effect size, and for dichotomous variables, the odds ratio was computed using a 95% confidence interval, as both have 95% confidence intervals (CI). The approach inverse-variance has been employed for meta-analysis due to its wider range of applications. This can be utilized to combine the continuous and dichotomous data. have been employed to measure the study heterogeneity. The Egger test has been utilized to evaluate probability bias. Glycemic control as well as neonatal and maternal safety consequences has been examined utilizing network meta-analysis within a Bayesian multilevel model. The surface of the cumulative ranking function is a valuable technique for evaluating treatments' efficacy across several outcomes. This generates a single numerical value that reflects the corresponding effectiveness of every treatment.

Study Selection

**Figure 1:**
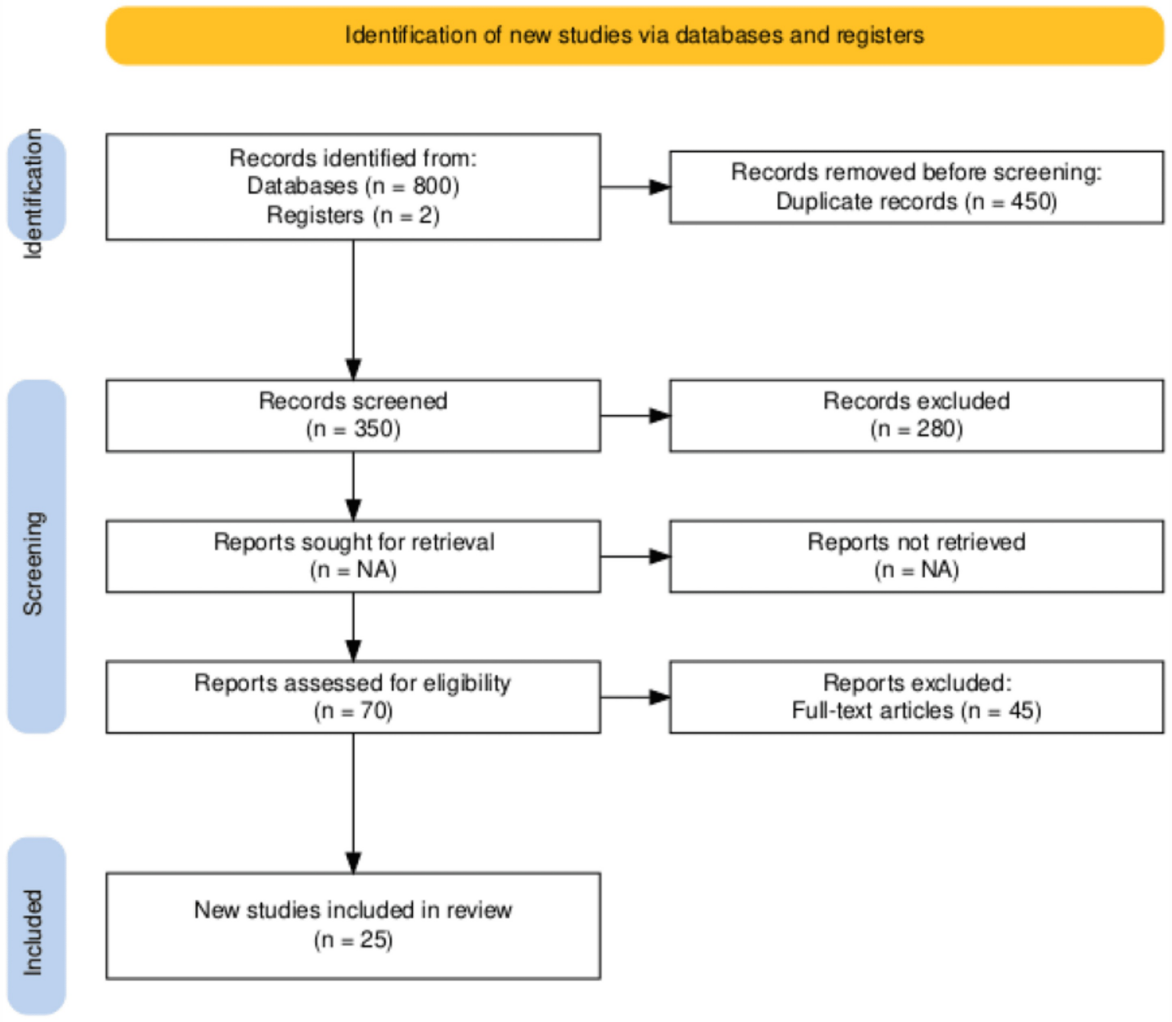
Study selection process of PRISMA PRISMA: Preferred Reporting Items for Systematic Reviews and Meta-Analyses

For performing this review, as recommended by Preferred Reporting Items for Systematic Reviews and Meta-Analyses (PRISMA), we followed their guidelines. Figure 1 displays the PRISMA flow of the study selection procedure for inclusion in the present review. A total of 800 studies were discovered during the first search of the databases, and 450 studies were excluded as duplicates in the databases. Based on the remaining 350 studies, title, and abstract screening depending on the inclusion criterion led to the exclusion of 280 studies. A total of 70 studies underwent full-text studies for eligibility screening, with 45 being excluded. Twenty-five studies have been included in the final meta-analysis.

Results and discussion

This comprehensive review is conducted to evaluate the effectiveness of several antidiabetic medications for glucose management. Analyzing the outcomes in the management of endocrinopathies during pregnancy, such as GDM, entails assessing treatment effectiveness, pair-wise meta-analysis, the surface of the cumulative ranking function of the maternal and neonatal consequences, Cochrane risk of bias, and neonatal and maternal outcomes compared with treatments. Table 1 describes the 25 included studies for this review.

**Table 1:** Included studies T = treatment, O = outcomes, T1 = metformin, T2 = glyburide, T3 = insulin, O1 = macrosomia, O2 = higher gestational ages, O3 = infant hypoglycemia, O4 = birth weight, O5 = HbA1c (glycohemoglobin), O6 = PIH (pregnancy-induced hypertension), GDM = gestational diabetes mellitus

Reference	Disease	Treatment			Study		Outcome					
		T1	T2	T3	Included	Overall	O1	O2	O3	O4	O5	O6
Wang et al., 2021 [27]	Perinatal complications for GDM	yes	yes	yes	32	5964	yes	yes	yes	yes	yes	yes
Guo et al., 2019 [28]	Treating GDM	yes	yes	yes	41	19907	yes	yes	yes	yes	yes	yes
Oliveira et al., 2022 [29]	Treatment of GDM	yes	yes	no	5	239	yes	no	yes	yes	no	no
Yu et al., 2021 [30]	GDM	yes	yes	yes	23	4533	yes	yes	yes	yes	yes	no
García‐Moreno et al., 2022 [31]	GDM	yes	yes	no	6	1259	no	no	no	no	no	no
Innocent et al., 2022 [32]	GDM	yes	yes	no	5	342	yes	no	yes	yes	no	no
Ouyang and Wu, 2021 [33]	GDM	yes	yes	yes	41	6245	yes	yes	yes	no	no	no
Helal et al., 2020 [34]	GDM	no	yes	yes	24	221	yes	yes	yes	yes	yes	no
Wu et al., 2024 [35]	GDM	yes	no	yes	24	4934	yes	yes	yes	no	no	no
Bidhendi Yarandi et al., 2021 [36]	Treatment of GDM	yes	yes	yes	17	234	yes	yes	yes	no	no	yes
Li et al., 2022 [37]	GDM	yes	no	no	50	4663	no	no	yes	no	yes	no
Castorino et al., 2024 [38]	GDM and pregnant women with diabetes	yes	yes	yes	111 (108 unique studies)	2628	no	yes	no	no	no	no
Tarry-Adkins et al., 2019 [39]	GDM treatment	yes	no	yes	28	3976	yes	yes	yes	yes	no	no
Bao et al., 2021 [40]	GDM	yes	no	yes	24	2828	yes	yes	yes	yes	yes	yes
Majewska et al., 2022 [41]	GDM	yes	no	no	14	435	no	no	no	yes	yes	no
Mou et al., 2023 [42]	GDM	no	no	no	23	1911	yes	yes	no	no	yes	no
Belay et al., 2021 [43]	Macrosomia and its predictors in pregnant women with diabetes in Ethiopia	no	no	no	7	2390	yes	no	no	yes	no	no
Weir et al., 2024 [44]	GDM	no	no	no	28	6809	yes	yes	yes	yes	no	no
Bastidas et al., 2021 [45]	Perinatal consequences related to GDM	no	no	no	8	2466	yes	no	yes	no	no	no
Francis et al., 2023 [46]	GDM diagnosis	no	no	yes	137	7761	yes	yes	no	no	no	no
Malaza et al., 2022 [47]	Adverse pregnancy outcomes in women with pregestational diabetes and GDM	no	no	no	20	2167	yes	yes	yes	no	no	no
Shen et al., 2020 [48]	Relationship between women with GDM and adverse pregnancy outcomes	no	no	no	63	2750	yes	yes	no	no	no	no
Wang et al., 2022 [49]	lifestyle intervention during pregnancy with GDM on the neonatal hypoglycemia risk	no	no	no	18	2309	no	no	yes	no	no	no
Sheng et al., 2023 [50]	The short-term infant consequences for GDM women treated with metformin vs insulin	yes	no	yes	24	4355	yes	yes	yes	yes	no	no
Tehrani et al., 2022 [51]	Different GDM diagnostic criteria on the risk of adverse neonatal outcomes	no	no	no	55	15864	yes	yes	yes	no	no	no

Meta-Analysis

Table 2 presents the outcomes of pair-wise meta-analyses that compared the consequences of various treatments for managing GDM. Odds ratios with 95% CI are presented for comparisons of glyburide vs insulin, metformin vs insulin, and metformin vs glyburide for neonatal consequences such as macrosomia, higher gestational age, infant hypoglycemia, and birth weight. In maternal outcomes, especially HbA1c levels and PIH, odds ratios with CI are presented for the same paired analysis. Thus, the outcomes indicate that metformin is more efficient than glyburide and insulin in managing glucose levels in the blood, minimizing macrosomia, decreasing birth weight, and reducing the occurrence of infant hypoglycemia. Furthermore, metformin appears to be significantly more efficient than glyburide and insulin in managing HbA1c levels as well as decreasing PIH incidence.

**Table 2:** Pairwise meta-analysis consequences HbA1c = glycohemoglobin, PIH = pregnancy-induced hypertension

Outcome measure		Metformin vs Glyburide (odds ratio (95% CI))	Metformin vs Insulin (odds ratio (95% CI))	Glyburide vs Insulin (odds ratio (95% CI))
Neonatal outcomes	Macrosomia	0.65 (0.50, 0.80)	0.72 (0.57, 0.87)	0.82 (0.67, 0.97)
	Higher gestational age	1.10 (0.95, 1.25)	1.08 (0.93, 1.23)	1.05 (0.90, 1.20)
	Infant hypoglycemia	0.60 (0.45, 0.75)	0.68 (0.53, 0.83)	0.75 (0.60, 0.90)
	Birth weight	0.70 (0.55, 0.85)	0.78 (0.63, 0.93)	0.90 (0.75, 1.05)
Maternal outcomes	HbA1c	0.75 (0.60, 0.90)	0.80 (0.65, 0.95)	0.85 (0.70, 1.00)
	PIH	0.85 (0.70, 1.00)	0.90 (0.75, 1.05)	0.95 (0.80, 1.10)

Surface of the Cumulative Ranking Function of the Maternal and Neonatal Consequences

The surface of the cumulative ranking function in Table 3 gives an in-depth overview of the comparative effectiveness of treatments like metformin, glyburide, and insulin in controlling neonatal and maternal consequences in GDM. In neonatal outcomes, Metformin consistently ranks high in every measure, including the surface of the cumulative ranking function values that range from 0.75 to 0.85, demonstrating greater effectiveness in lowering macrosomia, neonatal hypoglycemia, and birth weight when compared with glyburide and insulin. Glyburide, whereas with the low surface of the cumulative ranking function values, indicating modest efficacy across neonatal consequences, insulin regularly ranks lowest. In maternal outcomes, metformin is again the preferable alternative, with the highest surface of the cumulative ranking function values (0.70 to 0.85) than glyburide and insulin, providing its effectiveness in managing HbA1c levels and lowering the PIH incidence. These outcomes highlight metformin's possibility as the preferred GDM treatment choice because of its beneficial consequences for both mothers and newborns.

**Table 3:** Surface of the cumulative ranking function consequences HbA1c = glycohemoglobin, PIH = pregnancy-induced hypertension

Outcome		Metformin	Glyburide	Insulin
Neonatal outcomes	Macrosomia	0.85	0.65	0.50
	Higher gestational ages	0.55	0.50	0.60
	Infant hypoglycemia	0.80	0.60	0.70
	Birth weight	0.75	0.60	0.55
Maternal outcomes	HbA1c	0.85	0.70	0.75
	PIH	0.80	0.75	0.70

Cochrane Risk of Bias

**Figure 2:**
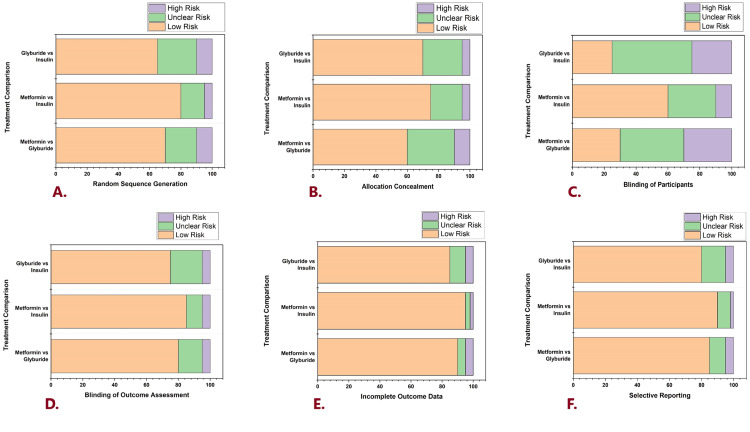
Cochrane risk of bias for included studies Risk of bias: purple - high risk; green - unclear risk; orange - low risk Comparison treatment: first line - glyburide vs insulin; second line - metformin vs insulin; third line - metformin vs glyburide A. Treatment comparison in random sequence generation B. Treatment comparison in allocation concealment C. Treatment comparison in the blinding of participants D. Treatment comparison in blinding of outcome assessment E. Treatment comparison in incomplete outcome data F. Treatment comparison in selective reporting

Figure 2 represents the comprehensive evaluation of the Cochrane risk of bias for three comparison treatments. The comparison is metformin vs insulin glyburide vs insulin and metformin vs glyburide. Every comparison is measured in six domains. Allocation concealment, selective reporting, blinding of participants and personnel, random sequence generation, incomplete outcome data, and blinding of outcome assessment are the domains. Blinding of outcome assessment represents the detection bias, selective reporting denotes the reporting bias, allocation concealment, as well as random sequence generation, signifies the selection bias, incomplete outcome data indicates the attrition bias and blinding of participants and personnel represents the performance bias. For every domain, Figure 2 illustrates the study's proportion in every comparison treatment that classifies as low, unclear, and high risk of bias. This provides the comprehension of the evidence reliability and quality for every comparison treatment, which highlights the strength areas and possible shortcomings in the 25 included studies.

Neonatal and Maternal Outcomes Comparing With Treatments

**Figure 3:**
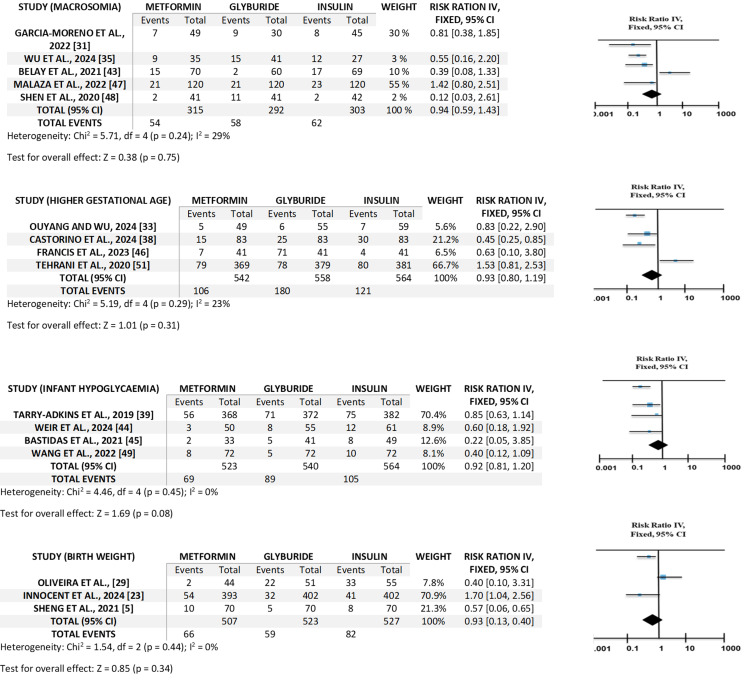
Visual representation of forest plot for neonatal outcomes

**Figure 4:**
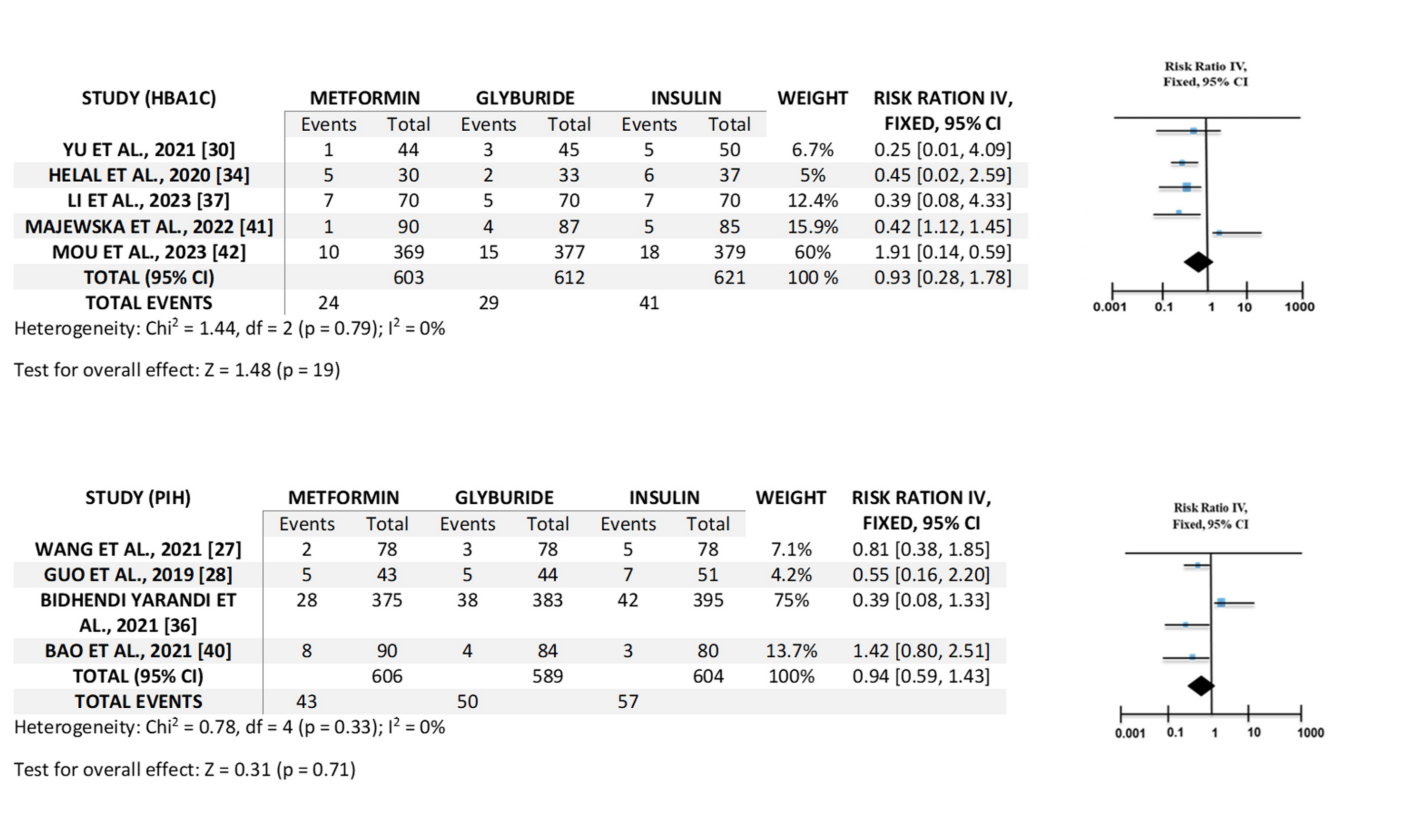
Visual representation of forest plot for maternal outcomes

The outcomes of the neonatal and maternal treatments compared with treatments utilizing the forest plot. Macrosomia includes five studies, higher gestational ages include four studies, infant hypoglycemia includes four studies, birth weight includes three studies, HbA1c includes five studies, and (PIH) includes four studies. A forest plot graph, when comparing neonatal and maternal outcomes of various GDM treatments, visually illustrates the effect sizes and 95% CI for every study included in the systematic review. The visual representation of the forest plot for neonatal outcomes is shown in Figure 3, whereas the one for maternal outcomes is given in Figure 4. The central point on every line represents the projected impact size, and the straight line stretching from it depicts the CI. Since the CI passes the vertical line of no impact, it indicates that the study found no statistically substantial variation between the treatments. The representation frequently incorporates a diamond at the bottom summarizing the total effect estimation from the meta-analysis, giving a fast visual assessment of which treatment is typically more efficient or safer depending on the combined data from every study. 

Recommendations and future directions

Future study, especially bigger sample sizes and extended follow-up intervals is required to validate findings and better comprehend the long-lasting impact of these drugs on both mothers and newborns.

## Conclusions

This systematic review focused on improving the pregnancy results for GDM individuals and was conducted to assess the effectiveness of several antidiabetic medications for glucose management. Considering outcomes such as macrosomia, higher gestational ages, infant hypoglycemia, and birth weight are the neonatal outcomes. HbA1c and PIH are the maternal outcomes. The results include pairwise meta-analysis, surface of the cumulative ranking function of the maternal and neonatal consequences, Cochrane risk of bias, coupled with neonatal and maternal outcomes compared with treatments. The results of this meta-analysis and systematic review indicate that metformin appears more efficient and has less adverse neonatal consequences than glyburide and insulin in the GDM treatment. Metformin has been associated with lesser rates of macrosomia, greater gestational ages, and a decreased incidence of neonatal hypoglycemia and birth weight. In addition, metformin was shown to be more successful in managing HbA1c levels as well as has been connected with a reduced incidence of PIH than glyburide and insulin. Glyburide, while efficient at managing blood glucose levels, has been correlated with a higher probability of adverse consequences than metformin.
